# Derivation and validation of an easy-to-compute trauma score that improves prognostication of mortality or the Trauma Rating Index in Age, Glasgow Coma Scale, Respiratory rate and Systolic blood pressure (TRIAGES) score

**DOI:** 10.1186/s13054-019-2636-x

**Published:** 2019-11-21

**Authors:** Atsushi Shiraishi, Yasuhiro Otomo, Shunsuke Yoshikawa, Koji Morishita, Ian Roberts, Hiroki Matsui

**Affiliations:** 1 Emergency and Trauma Center, Kameda Medical Center, 929, Higashicho, Kamogawa, Chiba 296-8602 Japan; 2 Trauma and Acute Critical Care Center, Tokyo Medical and Dental University Hospital of Medicine, 1-5-45, Yushima, Bunkyo-ku, Tokyo, 113-8510 Japan; 3 Clinical Trials Unit, London School of Hygiene & Tropical Medicine, Keppel Street, London, WC1E 7HT UK; 4 Department of Clinical Epidemiology and Health Economics, School of Public Health, The University of Tokyo, 7-3-1, Hongo, Bunkyo-ku, Tokyo, 113-8655 Japan; 5 Clinical Research Support Office, Kameda Medical Center, Kamogawa, Chiba 296-8602 Japan

**Keywords:** Trauma score, Revised Trauma Score, MGAP score, Prognostic accuracy study, Trauma registry, In-hospital mortality

## Abstract

**Background:**

Multiple trauma scores have been developed and validated, including the Revised Trauma Score (RTS) and the Mechanism, Glasgow Coma Scale, Age, and Arterial Pressure (MGAP) score. However, these scores are complex to calculate or have low prognostic abilities for trauma mortality. Therefore, we aimed to develop and validate a trauma score that is easier to calculate and more accurate than the RTS and the MGAP score.

**Methods:**

The study was a retrospective prognostic study. Data from patients registered in the Japan Trauma Databank (JTDB) were dichotomized into derivation and validation cohorts. Patients’ data from the Clinical Randomisation of an Antifibrinolytic in Significant Haemorrhage-2 (CRASH-2) trial were assigned to another validation cohort. We obtained age and physiological variables at baseline, created ordinal variables from continuous variables, and defined integer weighting coefficients. Score performance to predict all-cause in-hospital death was assessed using the area under the curve in receiver operating characteristics (AUROC) analyses.

**Results:**

Based on the JTDB derivation cohort (*n* = 99,867 with 12.5% mortality), the novel score ranged from 0 to 14 points, including 0–2 points for age, 0–6 points for the Glasgow Coma Scale, 0–4 points for systolic blood pressure, and 0–2 points for respiratory rate. The AUROC of the novel score was 0.932 for the JTDB validation cohort (*n* = 76,762 with 10.1% mortality) and 0.814 for the CRASH-2 cohort (*n* = 19,740 with 14.6% mortality), which was superior to RTS (0.907 and 0.808, respectively) and MGAP score (0.918 and 0.774, respectively) results.

**Conclusions:**

We report an easy-to-use trauma score with better prognostication ability for in-hospital mortality compared to the RTS and MGAP score. Further studies to test clinical applicability of the novel score are warranted.

## Background

A prognostic score aims to provide standardized severity metrics for a specific medical condition and to stratify patients into groups according to the probability of the prognosis [1]. A trauma score could be useful in improving the quality of care and in assisting with prognostication in the patient group [2]. In this context, stratification by a trauma score classifies patients into low, moderate, or high risk for outcome [3] and can be applicable as a triage tool in disasters, mass casualty incidents, or military settings [4].

Over time, multiple trauma scores have been developed. These include trauma scores based on the patient’s information obtained in the early trauma care, anatomical trauma scores based on the distribution and severity of the injury, and combined trauma scores based on early, anatomical, and laboratory information [2]. Trauma scores in the early trauma care typically include several predictors, such as age, mechanism of injury, and physiological status; such scores include the Revised Trauma Score (RTS) [5, 6] and the Mechanism, Glasgow Coma Scale (GCS), Age, and Arterial Pressure (MGAP) score [7]. To calculate the RTS, GCS, systolic blood pressure, and respiratory rate are assigned one of five categories from 0 to 4 points; this score is multiplied by the weighting coefficients of 0.9368 for GCS, 0.7326 for systolic blood pressure, and 0.2908 for respiratory rate. The total of these three items is then calculated [6]. Although using RTS in early trauma care provides an acceptable prediction of trauma mortality [6, 8, 9], its computation is complex, and its weighting coefficients are reportedly out of date [10–12]. Therefore, the MGAP score was developed as the simple sum of the categorized values without using the weighting coefficients [7]. Although the MGAP score is easy to compute, its prognostic ability for trauma mortality is not superior to that of the RTS [9].

This study aimed to develop and validate a novel, easy-to-calculate trauma score with improved prognostication ability for trauma mortality compared with the RTS and MGAP scores.

## Methods

### Study design and setting

This retrospective prognostic study aimed to develop and validate a prognostic model for in-hospital mortality in adult trauma patients. Reporting of this study adhered to the Transparent Reporting of a Multivariable Prediction Model for Individual Prognosis or Diagnosis (TRIPOD) guideline [13] and was approved by the medical ethics committee of the Tokyo Medical and Dental University (reference number 2192).

Data was obtained from patients’ all-available data of the nationwide trauma registry in Japan (the Japan Trauma Databank [JTDB]) from the year 2004 to 2015 and that of the Clinical Randomisation of an Antifibrinolytic in Significant Haemorrhage-2 (CRASH-2) trial from the year 2005 to 2010 [14–16]. The JTDB was established in 2004 and involved 256 hospitals in Japan by 2015 [14, 17]. Participating hospitals in the JTDB voluntarily register trauma patients with an Injury Severity Score (ISS) ≥ 9 and burn patients and include information on demographics, the situation and mechanism of injury, the physiological status before and after arrival at the emergency room (ER), procedures before and after arrival at the ER, surgeries in the ER and/or operation theater, the Abbreviated Injury Scale (AIS) value, ISS, death, and length of hospitalization [14, 17].

CRASH-2 was a randomized controlled trial aimed at assessing the effects of tranexamic acid in bleeding trauma patients in 40 countries, mainly developing countries [15, 16]. CRASH-2 also collected the following data from trauma patients: demographics, physiological status after arrival at the ER, death during hospitalization, and length of hospitalization [15, 16].

### Selection of participants

The present study included patients with documented blunt or penetrating trauma, but without a burn. The study exclusion criterion was defined as the exclusion of patients aged < 16 years or unreported. This exclusion criterion was determined based on the available information before the outlier removal and multiple imputation and applied to the multiply imputed datasets after the outlier removal and multiple imputation.

### Measurements

We randomly dichotomized the JTDB cases into derivation and validation cohorts according to a unique identification number provided to each participating institute. Patients from institutes with an even unique identification number were allocated to the JTDB derivation cohort, and the remaining patients were allocated to the JTDB validation cohort. All patients from CRASH-2 were allocated to the other validation cohort. The JTDB derivation cohort was used for score development, and the JTDB and CRASH-2 validation cohorts were used for validation of the developed scoring system.

Study predictor variables included the mechanism of injury (blunt or penetrating) as a nominal variable and age (years), respiratory rate (1/min), systolic blood pressure (mmHg), and GCS as the continuous variables. These variables were obtained at the initial contact with a trauma patient in the ER and upon entering the study according to the JTDB and CRASH-2 definition, respectively. Other variables (e.g., prehospital vital signs or the ISS) were also included and used for multiple imputation, sensitivity analyses, and/or explanatory subgroup analysis.

To preserve statistical power and appropriately assess the association between predictor variables and outcome while minimizing selection bias, the JTDB derivation cohort datasets underwent a 2-step data preparation before statistical analyses. The validation datasets did not undergo this process to simulate “real-world” conditions. The first step included the detection and removal of outliers from the numeric variables, using robust linear regression analyses [18], followed by multiple imputation by chained equation (MICE) with 20 iterations that generated 25 datasets with imputed missing values [19]. The Box-Cox transformation method was used to transform the distribution of all numerical variables into normal distribution prior to imputation, and to transform them back into the original distribution after imputation. For GCS, MICE imputed 3 different elements of GCS (eye, verbal, and motor responses) as ordered variables separately. Verbal GCS subscore was recorded as if the patient was intubated. The total GCS was recalculated as the sum of the 3 different elements of GCS after imputation. An AIS code of 9 in any section was regarded as a missing value and was multiply imputed.

### Outcomes

We defined the study reference standard as in-hospital death from any cause.

### Analysis

A logistic regression analysis, with age and physiological status including GCS, systolic blood pressure, and respiratory rate on arrival at the ER as explanatory variables, was used to predict in-hospital mortality. Relations between several predictors and outcomes did not assure linear and monotonic function. Therefore, in addition to the ordinal logistic regression analysis, a multivariable generalized additive model was used for visual assessment of non-linear and non-monotonic functions between the predictors and study outcome [20]. In reference to the magnitudes of the regression coefficients of the logistic regression analysis and the results of the generalized additive model, the ranges for all predictors were partitioned, assigned simple integers, and included in the prediction model, which was then developed into a novel trauma score. A detailed method for partitioning numeric data into integerized score points for the novel score is provided (Additional file 1).

The primary and secondary analyses compared the prediction of in-hospital mortality with the novel score versus RTS or MGAP score, in both the JTDB validation and the CRASH-2 cohorts. The primary analysis involved the use of receiver operating characteristic (ROC) analyses to evaluate the prognostic accuracy of the scores in terms of the area under the ROC curve (AUROC). A Hosmer-Lemeshow plot was used to assess the calibration of the predicted and observed in-hospital mortality, estimated on both the JTDB validation and CRASH-2 cohorts. The two best thresholds of tested scores to predict in-hospital mortality were estimated in the JTDB derivation cohort using Youden’s index and a sensitivity > 0.9. These thresholds were further used to determine sensitivity, specificity, and positive and negative predictive values of the scores predicting in-hospital mortality in the ROC curves in the primary analysis of validation cohorts. The required sample size for the study’s primary analysis was estimated based on the parameters obtained from the ROC analysis, comparing the novel score to RTS or MGAP score in the JTDB derivation cohort, and the given power of 0.8 and *P* value of 0.025, after Bonferroni modification of multiple comparisons [21]. The secondary analyses included partial AUROC analysis with restriction for a sensitivity of ≥ 0.9 and a reclassification improvement analysis. Integration of the point estimation and variances across the multiply imputed datasets were based on a 20,000-time bootstrapping in the ROC, partial ROC, and reclassification improvement analyses or Rubin’s rule, in all other analyses [19].

To test the robustness of the primary analysis, sensitivity analyses to reassess the primary analysis were performed on the validation datasets after outlier removal and multiple imputation and on validation datasets where the mechanism of trauma and physiological variables were imputed as “blunt” and normal values, respectively. To test for the applicability of scores in prehospital settings, a separate sensitivity analysis was performed using prehospital variables instead of hospital variables. Furthermore, explanatory subgroup analyses assessed the prediction for in-hospital mortality with scores in the subgroups stratified by age.

All statistical analyses were performed using “R 3.5.1” for statistical computing (R Foundation for Statistical Computing, Vienna, Austria) with several add-on packages.

## Results

### Characteristics of patients enrolled in the study

**Fig. 1:**
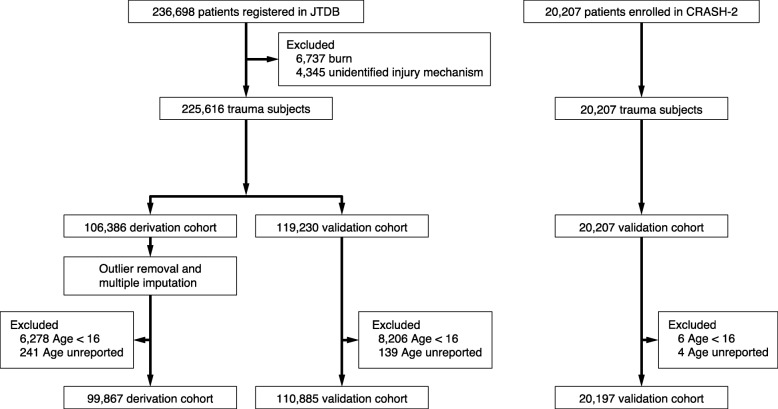
Study participant selection tree. Outlier removal from numerical variables and multiple imputation was applied after the selection of trauma subjects in the JTDB derivation cohort. Exclusion criteria for the JTDB derivation cohort were determined based on patient background characteristics before outlier removal and multiple imputation and were applied after outlier removal and multiple imputation. JTDB, the Japan Trauma Databank; CRASH-2 Clinical Randomisation of an Antifibrinolytic in Significant Haemorrhage-2

This study selected 210,752 cases from among 225,616 trauma patients from the JTDB (JTDB derivation cohort, 107 hospitals, *n* = 99,867; JTDB validation cohort, 114 hospitals, *n* = 110,885; Fig. 1). Similarly, 20,197 of 20,207 patients from the CRASH-2 were assigned to the CRASH-2 validation cohort (Fig. 1). Patients in the JTDB derivation and validation cohorts were similar, whereas patients in the CRASH-2 cohort were younger, more frequently had a penetrating injury, and were more hemodynamically unstable, with lower systolic blood pressure and a higher heart rate at presentation than those in the JTDB cohorts (Table 1, details provided in Additional file 2: Table S1).

**Table 1:** Baseline characteristics of the study population

Variables	JTDB derivation cohort	JTDB validation cohort	CRASH-2 validation cohort
Number of subjects	99,867	110,885	20,197
Multiple imputation	Yes	No	No
Sex, *n* (%)			
Male	63,177 (63.3)	68,132 (61.4)	16,927 (83.8)
Female	36,690 (36.7)	42,727 (38.5)	3269 (16.2)
Missing data	0 (0.0)	26 (0.0)	1 (0.0)
Age, years [range]	61 [39, 76]	63 [41, 78]	30 [24, 43]
Missing data, *n* (%)	0 (0.0)	0 (0.0)	0 (0.0)
Type of trauma, *n* (%)			
Blunt injury	95,943 (96.1)	104,110 (93.9)	11,184 (55.4)
Penetrating injury	3745 (3.7)	3658 (3.3)	6549 (32.4)
Blunt and penetrating injury	178 (0.2)	158 (0.1)	2464 (12.2)
Missing data	1 (0.0)	2959 (2.7)	0 (0.0)
Physiological signs on arrival at an emergency department			
Systolic blood pressure, mmHg [range]	132 [110, 155]	135 [115, 156]	95 [80, 110]
Missing data, *n* (%)	0 (0.0)	4014 (3.6)	318 (1.6)
Heart rate, beats/min [range]	81 [70, 96]	82 [70, 95]	105 [90, 120]
Missing data, *n* (%)	0 (0.0)	5594 (5.0)	137 (0.7)
Respiratory rate, rate/min [range]	20 [16, 24]	20 [16, 24]	22 [20, 26]
Missing data, *n* (%)	0 (0.0)	17,539 (15.8)	191 (0.9)
Glasgow Coma Scale	15 [13, 15]	15 [14, 15]	15 [11, 15]
Missing data, *n* (%)	0 (0.0)	9884 (8.9)	23 (0.1)
Traumatic brain injury, *n* (%)	30,666 (30.7)	30,893 (27.9)	Not available
Missing data, *n* (%)	0 (0.0)	7515 (6.8)	Not available
Injury Severity Score	13 [9, 22]	10 [9, 20]	Not available
Missing data, *n* (%)	0 (0.0)	8226 (7.4)	Not available

Categorical variables are displayed as count with percentage, and continuous variables are displayed as median with 25th–75th interquartile range. Traumatic brain injury was defined as having the abbreviated injury scale of ≥ 3 on the head region

*JTDB* Japan Trauma Databank, *CRASH-2* Clinical Randomisation of an Antifibrinolytic in Significant Haemorrhage-2, *mmHg* millimeter of mercury

### Main results

#### Score development

**Fig. 2:**
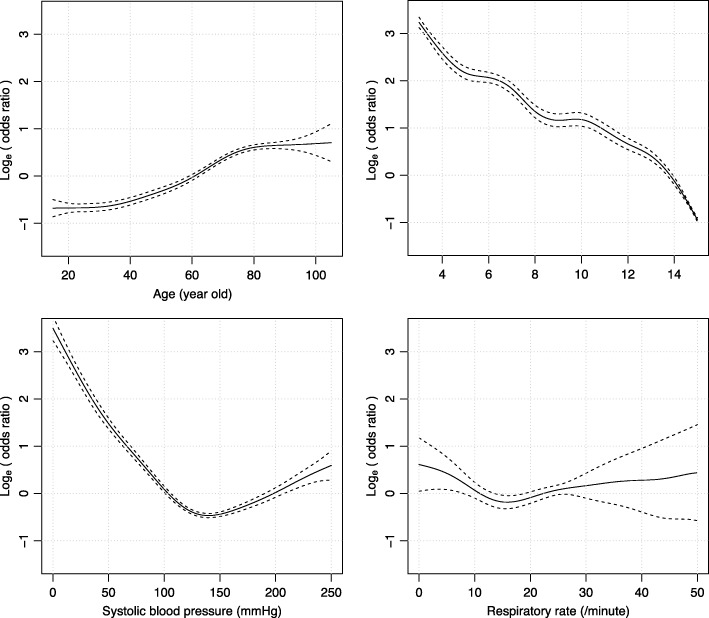
Non-linear association of physiological status variables and in-hospital mortality in a multivariable generalized additive model. A multivariable generalized additive model (GAM), which included age, systolic blood pressure, Glasgow Coma Scale, and respiratory rate on arrival at the emergency department as explanatory variables and in-hospital mortality as a response variable on the JTDB derivation cohort, estimated the non-linear regression curve with a logit link function. GAM plots were estimated and integrated across multiply imputed datasets with a number of knots of 10; line, point estimate; dotted line, upper and lower margin of 95% confidence interval

In the JTDB derivation cohort, 12,473/99,867 (12.5%) patients died in the hospital. The logistic regression generalized additive model used to predict in-hospital mortality for the JTDB derivation cohort demonstrated approximately monotonic positive and negative correlations for age and GCS, respectively, and curvilinear U-shaped correlations with respiratory rate and systolic blood pressure (Fig. 2). Based on the logistic regression analysis to predict in-hospital mortality, after assignment of simple integers to the categorized ranges in the predictor values, we defined a novel score that summed these 4-digit integers (the Trauma Rating Index in Age, Glasgow Coma Scale, Respiratory rate and Systolic blood pressure [TRIAGES] score, Additional file 1). The TRIAGES score ranges from 0 to 14 points: 0–2 points for age, 0–6 points for GCS, 0–2 points for respiratory rate, and 0–4 points for systolic blood pressure (Table 2, Additional file 3: Table S2). The required sample size for the test validation was estimated at 8237 or 13,623 for the comparison of the TRIAGES score to RTS or MGAP score, respectively.

**Table 2:** Predictors at presentation associated with in-hospital death in the Japan Trauma Databank derivation cohort

Predictors	Beta [95%CI]	Variance infraction factor	*P* value	Integerized score point
Intercept	− 5.00 [− 5.10, − 4.90]		< 0.001	
Age, years				
16–54	Reference			0
55–74	0.70 [0.62, 0.78]	1.45	< 0.001	1
75+	1.13 [1.05, 1.21]	1.53	< 0.001	2
Glasgow Coma Scale				
3	4.19 [4.07, 4.31]	1.82	< 0.001	6
4	3.63 [3.46, 3.80]	1.25	< 0.001	5
5–7	2.91 [2.80, 3.02]	1.64	< 0.001	4
8–11	2.12 [2.01, 2.23]	1.56	< 0.001	3
12–13	1.43 [1.31, 1.55]	1.45	< 0.001	2
14	0.86 [0.75, 0.98]	1.49	< 0.001	1
15	Reference			0
Respiratory rate, 1/min				
0–3	1.63 [1.42, 1.84]	1.42	< 0.001	2
4–11	0.54 [0.37, 0.71]	1.03	< 0.001	1
12–27	Reference			0
28+	0.56 [0.48, 0.64]	1.07	< 0.001	1
Systolic blood pressure, mmHg				
0–49	2.66 [2.52, 2.80]	1.37	< 0.001	4
50–79	1.31 [1.19, 1.42]	1.05	< 0.001	2
80–99	0.67 [0.56, 0.77]	1.05	< 0.001	1
100–199	Reference			0
200+	0.58 [0.45, 0.71]	1.03	< 0.001	1

Continuous predictor variables are categorized based on the regression coefficient (beta) estimated using logistic regression analysis. Integerized score points were assigned as every 0.67 of the magnitude of beta

*95%CI* 95% confidence interval, *mmHg* millimeter of mercury

#### Score validation

In the JTDB validation and CRASH-2 cohorts, 9877/97,428 (10.1%) and 3085/20,197 (15.3%) patients died in the hospital, respectively. From the AUROC analysis, the TRIAGES score showed the best prediction of in-hospital mortality for both the JTDB validation and CRASH-2 cohorts (Table 3, Additional file 4: Figure S1). The TRIAGES score also showed the best calibration of predicted and observed in-hospital mortality in terms of the Pearson chi-square statistic estimated for both the JTDB validation and CRASH-2 cohorts (Additional file 5: Figure S2). For the tested scores across the validation cohorts, sensitivity, specificity, and positive and negative predictive values at the selected thresholds were also assessed (Additional file 6: Table S3). In secondary analyses, the partial ROC analysis demonstrated that the TRIAGES score had the best partial AUROC if the given sensitivity was restricted to > 0.9 (Table 3). Another secondary analysis or a reclassification improvement analysis demonstrated that the TRIAGES score improved the reclassification, compared with both the RTS for the CRASH-2 cohort and the MGAP score for the JTDB validation and CRASH-2 cohorts (Table 3). Results of sensitivity analyses to reassess the primary analysis in multiply imputed or single-imputed-by-the-best-value datasets were consistent with those of the primary analyses (Table 3). Sensitivity analysis to assess the prediction of the outcome using prehospital variables was also consistent with that of the primary analysis (Table 3). In the explanatory subgroup analyses stratified by age groups, the prognostic accuracy of the tested scores was relatively worse in elderly patients in the JTDB validation cohort (Additional file 7: Table S4). This association was most obvious for RTS.

**Table 3:** Comparisons of prognostic accuracy and reclassification improvement based on tested scores

Analyses	Metrics of score performance			Bootstrap comparisons			
	TRIAGES score	RTS	MGAP score	TRIAGES score versus RTS		TRIAGES score versus MGAP score	
				Difference [95%CI]	*P*	Difference [95%CI]	*P*
Primary analysis							
Area under curve							
JTDB validation cohort (*n* = 76,762)	0.932	0.907	0.918	0.025 [0.023, 0.028]	< 0.001	0.014 [0.012, 0.016]	< 0.001
CRASH-2 cohort (*n* = 19,740)	0.814	0.808	0.774	0.006 [0.001, 0.011]	0.010	0.040 [0.033, 0.047]	< 0.001
Sensitivity analyses for the primary analysis							
Area under curve							
Multiple imputation							
JTDB validation cohort (*n* = 110,884)	0.917	0.894	0.902	0.023 [0.020, 0.025]	< 0.001	0.015 [0.013, 0.017]	< 0.001
CRASH-2 cohort (*n* = 20,197)	0.816	0.810	0.776	0.006 [0.002, 0.011]	0.006	0.040 [0.034, 0.046]	< 0.001
Single imputation by the best value							
JTDB validation cohort (*n* = 97,248)	0.911	0.889	0.899	0.022 [0.019, 0.024]	< 0.001	0.012 [0.009, 0.014]	< 0.001
CRASH-2 cohort (*n* = 20,197)	0.813	0.806	0.776	0.006 [0.002, 0.011]	0.006	0.037 [0.031, 0.043]	< 0.001
Prehospital variables							
JTDB validation cohort (*n* = 110,885)	0.858	0.786	0.833	0.072 [0.066, 0.078]	< 0.001	0.025 [0.021, 0.030]	< 0.001
Secondary analyses							
Partial area under curve (sensitivity ≥ 0.9)							
JTDB validation cohort (*n* = 110,885)	0.764	0.652	0.722	0.112 [0.102, 0.121]	< 0.001	0.042 [0.033, 0.050]	< 0.001
CRASH-2 cohort (*n* = 20,197)	0.629	0.592	0.560	0.037 [0.026, 0.048]	< 0.001	0.069 [0.057, 0.081]	< 0.001
Net reclassification improvement							
JTDB validation cohort (*n* = 110,885)				0.465 [0.441, 0.489]	< 0.001	0.422 [0.396, 0.447]	< 0.001
CRASH-2 cohort (*n* = 20,197)				0.230 [0.177, 0.312]	0.004	0.239 [0.176, 0.318]	< 0.001

Point estimation with 95%CI and *P* values of the metrics of score performance were computed by bootstrap estimation repeated 20,000 times (800 times per each dataset if bootstrapping was performed on multiply imputed datasets)

*TRIAGES* Trauma Rating Index in Age, Glasgow Coma Scale, Respiratory rate and Systolic blood pressure; *RTS* Revised Trauma Score; *MGAP* Mechanism, Glasgow Coma Scale, Age, and Arterial Pressure; *95%CI* 95% confidence interval; *JTDB* Japan Trauma Databank; *CRASH-2* Clinical Randomisation of an Antifibrinolytic in Significant Haemorrhage-2

## Discussion

Our novel trauma score is easy to calculate and improves the predictive accuracy compared with the RTS and MGAP scores, based on AUROC analyses, partial AUROC analysis, and reclassification improvement analysis, for both the JTDB validation cohort and CRASH-2 cohort.

The Trauma Injury Severity Score (TRISS) and Revised Injury Severity Classification version II (RISC-II) integrate the early information and delayed information from radiological images and/or laboratory tests to achieve the most accurate prognostication [22, 23]. However, the collection of late information requires considerable delays during this critical period for trauma patients. Furthermore, late information regarding radiological images usually involves a CT scan; therefore, a combined trauma score is often missed or miscalculated if a CT scan lacks. Trauma scores in the early trauma care are calculated based on the patients’ age, mechanisms of injury, and physiological status, for which information is easily collected during early trauma care and within minutes after initial patient contact with fewer missing [5–7, 9]. This facilitates the use of the score by healthcare professionals and is expected to be useful for triage in disasters, mass casualty incidents, and military settings [4]. In addition, the ease of calculating the score is an indispensable feature of a trauma score [10, 12]. Calculation of complex equations using weighted coefficients generally requires electrical devices that narrow the applicability to various situations. In contrast, simplifying the score design may potentially reduce the accuracy of outcome prediction.

The RTS is not easy to use at the bedside or in a prehospital setting because of its difficult categorization and relative complexity [5, 6]. Categorization of the parameters to compute the RTS requires discrimination between 0 and 1–49 mmHg for systolic blood pressure and between 0 and 1–5 breaths/min for respiratory rate which can be difficult to complete at the bedside within a period of seconds. Moreover, many modern prediction scores no longer use weighting coefficients with a decimal point, as they can complicate score computation in the absence of technological assistance. The TRIAGES and MGAP scores both avoid the discrimination of systolic blood pressure and respiratory rate values close to 0 and the use of weighting coefficients with a decimal point.

Generally, in trauma care, trauma scores can be adjusted for trauma severity for outcome research and international or institutional benchmarking [2]. Improving the accuracy and usability of trauma scores could also improve the quality of observational studies. In specific situations, such as disaster medicine or military medicine, a good trauma triage tool should allow the extraction of patients with less severe trauma, who need urgent treatment, as well as dying trauma patients, who no longer require treatment. Fulfilling these requirements can help develop a good triage trauma score, capable of extracting the smaller portion of the target population whose prognosis is modifiable, thereby saving medical resources.

The current study has several limitations. First, the use of a trauma database consisting of retrospectively recruited, non-consecutive trauma patients (JTDB) is not ideal for developing a prediction model. Second, the advantages of the TRIAGES score including simplicity and good prognostic accuracy are theoretically suitable in disaster or military settings, however not tested in the present study. Third, the use of in-hospital mortality as the study outcome is not appropriate for predicting long-term mortality in trauma patients. Fourth, the CRASH-2 dataset, which consists of participants outside of Japan, acted as an external validation dataset for this model to test the external validity of the trauma scores. However, CRASH-2 was a randomized controlled trial involving hemodynamically unstable trauma patients; therefore, the study did not include patients with critical or less-severe trauma. To account for the drawback of the CRASH-2 cohort as an external validation cohort, we also used a JTDB validation cohort.

## Conclusions

A novel trauma score that is easy to calculate and that improves prognostication was developed and validated.

## Supplementary information

**Additional file 1.** Detailed methods partitioning numeric data into integerized score points.

**Additional file 2:** **Table S1.** Study variables included in outlier removal and multiple imputation.

**Additional file 3:** **Table S2.** Partitioning with assigned score values of physiological parameters included in the tested trauma scores.

**Additional file 4:** **Figure S1.** Comparison of prognostic accuracies for in-hospital mortality in the studied trauma scores.

**Additional file 5:** **Figure S2.** Calibration plots of the studied trauma scores.

**Additional file 6:** **Table S3.** Comparisons of diagnostic indices across the validation cohorts.

**Additional file 7:** **Table S4.** Comparison of diagnostic accuracy of the tested scores stratified by age.

## Data Availability

The datasets used and/or analyzed during the current study are available from the corresponding author on reasonable request.

## Abbreviations

AIS: Abbreviated Injury Scale
AUROC: Area under the curve in receiver operating characteristics
CRASH-2: Clinical Randomisation of an Antifibrinolytic in Significant Haemorrhage-2
CT: Computed tomography
ER: Emergency room
GCS: Glasgow Coma Scale
ISS: Injury Severity Score
JTDB: Japan Trauma Databank
MGAP: Mechanism, Glasgow Coma Scale, Age, and Arterial Pressure
MICE: Multiple imputation by chained equation
RISC-II: Revised Injury Severity Classification version II
ROC: Receiver operating characteristics
RTS: Revised Trauma Score
TRIAGES: Trauma Rating Index in Age, Glasgow Coma Scale, Respiratory rate and Systolic blood pressure
TRISS: Trauma Injury Severity Score
