# Supplementary material for: Derivation and validation of an easy-to-compute trauma score that improves prognostication of mortality or the Trauma Rating Index in Age, Glasgow Coma Scale, Respiratory rate and Systolic blood pressure (TRIAGES) score
Source: Crit Care. 2019 Nov 21;23:365. doi: 10.1186/s13054-019-2636-x (PMC6868841; doi:10.1186/s13054-019-2636-x)
Supplement: Supplementary file 1 — Additional file 1. Detailed methods partitioning numeric data into integerized score points. [file 13054_2019_2636_MOESM1_ESM.pdf]

Additional File 1.
Detailed methods partitioning numeric data into integerized score points

The first author (AS) conducted a semi-automatic determination of point system of the TRIAGES score based on logistic regression analysis including hospital mortality as a binomial response variable, and age and physiologic variables as continuous predictor variables. Data utilized in this analysis was the multiply-imputed JTDB derivation dataset. R scripts and logs were attached to help understanding details of the analyses.

Step 1. Initial partitioning

Each predictor variables were partitioned into equal intervals which included age categories for every 5 years (i.e. ..., 30 to 34, 35 to 39, 40 to 44, ...), the Glasgow Coma Scale (GCS) for each 1 (i.e. ..., 13, 14 and 15), respiratory rate for every 4 /minute (i.e. 0 to 3, 4 to 7, 8 to 11, ...) and systolic blood pressure for every 10 mmHg (i.e. ..., 90 to 99, 100 to 109, 110 to 119, ... To avoid unrealistic partitioning around 0 mmHg in the systolic blood pressure (discrimination between 0 and 1 to 49 mmHg such as in the Revised Trauma Score is practically difficult), systolic blood pressure of 0 to 49 mmHg was further integrated into the 1 category.

Step 2. Determination of initial reference category

A logistic regression analysis estimated regression coefficients of each categorized predictors for hospital mortality. The category with the least mortality in each predictor variable (age 20 to 24 year old, GCS 15, respiratory rate 12 to 15 /minute and systolic blood pressure 130 to 139 mmHg) was selected as the initial reference category.

R script and log for the step 1 and 2

```
> # An R script for the step 1 and 2
> # Following scripts generated a log of logistic regression
> # results on the multiply-imputed JTDB derivation datasets.
> fit<-
+   lapply(
+     # Number of multiply-imputed dataset was 25 (N.impute).
+     1:N.impute,
+     function(i){
+       # R function for generalized linear model (glm).
```

```
+      glm(
+        # Study outcome was defined as in-hospital mortality
+        I(DischargeTo=="Die")~
+          # Age was initially partitioned for every 5 years
+          I(Age.I==3)+I(Age.I==4)+I(Age.I==6)+I(Age.I==7)+
+          I(Age.I==8)+I(Age.I==9)+I(Age.I==10)+I(Age.I==11)+
+          I(Age.I==12)+I(Age.I==13)+I(Age.I==14)+I(Age.I==15)+
+          I(Age.I==16)+I(Age.I==17)+I(Age.I>=18)+
+          # The GCS was initially used as raw value
+          I(GCS==3)+I(GCS==4)+I(GCS==5)+I(GCS==6)+I(GCS==7)+
+          I(GCS==8)+I(GCS==9)+I(GCS==10)+I(GCS==11)+I(GCS==12)+
+          I(GCS==13)+I(GCS==14)+
+          # Respiratory rate (RR) was initially partitioned
+          # for every 4 /minute
+          I(RR.I==0)+I(RR.I==1)+I(RR.I==2)+I(RR.I==4)+
+          I(RR.I==5)+I(RR.I==6)+I(RR.I==7)+I(RR.I==8)+
+          I(RR.I==9)+I(RR.I==10)+I(RR.I==11)+I(RR.I==12)+
+          I(RR.I==13)+I(RR.I>=14)+
+          # Systolic blood pressure (SBP) was initially
+          # partitioned for every 10 /mmHg
+          I(SBP.I %in% c(0:4))+I(SBP.I==5)+I(SBP.I==6)+
+          I(SBP.I==7)+I(SBP.I==8)+I(SBP.I==9)+I(SBP.I==10)+
+          I(SBP.I==11)+I(SBP.I==12)+I(SBP.I==14)+I(SBP.I==15)+
+          I(SBP.I==16)+I(SBP.I==17)+I(SBP.I==18)+I(SBP.I==19)+
+          I(SBP.I==20)+I(SBP.I==21)+I(SBP.I==22)+I(SBP.I==23)+
+          I(SBP.I==24)+I(SBP.I>=25),
+        # Using logit binomial function as a link function.
+        family=binomial(link="logit"),
+        # Data was multiply imputed derivation datasets
+        # (TRIAGES.d).
+        data=TRIAGES.d[[i]])})
>
> # Integration of the results on multiply-imputed datasets.
> summary(
+   mice::pool(
+     mice::as.mira(
+       fit)))[,c("estimate","std.error","p.value")]
```

A R log for the step 1 and 2.

	estimate	std.error	p.value
(Intercept)	-5.471687828	0.11807504	0.000000e+00
I(Age.I == 3)TRUE	0.005356756	0.12320335	9.653204e-01
I(Age.I == 4)TRUE	0.005576854	0.11249299	9.604617e-01
I(Age.I == 6)TRUE	0.045222272	0.12253878	7.121003e-01
I(Age.I == 7)TRUE	0.025355182	0.11635053	8.274943e-01
I(Age.I == 8)TRUE	0.213006769	0.11825485	7.168563e-02
I(Age.I == 9)TRUE	0.354801815	0.11392443	1.847320e-03
I(Age.I == 10)TRUE	0.355520890	0.11250482	1.580925e-03
I(Age.I == 11)TRUE	0.483593379	0.10715023	6.439155e-06
I(Age.I == 12)TRUE	0.716458585	0.10263073	3.069989e-12
I(Age.I == 13)TRUE	0.889596544	0.09880990	0.000000e+00
I(Age.I == 14)TRUE	1.105185856	0.09840356	0.000000e+00
I(Age.I == 15)TRUE	1.252688119	0.09581246	0.000000e+00
I(Age.I == 16)TRUE	1.270213675	0.09741661	0.000000e+00
I(Age.I == 17)TRUE	1.310835926	0.10082878	0.000000e+00
I(Age.I >= 18)TRUE	1.307180028	0.10997897	0.000000e+00
I(GCS == 3)TRUE	4.177605714	0.06037281	0.000000e+00
I(GCS == 4)TRUE	3.613970681	0.08633317	0.000000e+00
I(GCS == 5)TRUE	2.774789063	0.10259153	0.000000e+00
I(GCS == 6)TRUE	3.131592947	0.06968147	0.000000e+00
I(GCS == 7)TRUE	2.678917489	0.07542786	0.000000e+00
I(GCS == 8)TRUE	2.322367217	0.09134397	0.000000e+00
I(GCS == 9)TRUE	2.050309788	0.09669868	0.000000e+00
I(GCS == 10)TRUE	2.144156054	0.08948680	0.000000e+00
I(GCS == 11)TRUE	1.869376631	0.09193156	0.000000e+00
I(GCS == 12)TRUE	1.605584647	0.08979723	0.000000e+00
I(GCS == 13)TRUE	1.316771430	0.07091174	0.000000e+00
I(GCS == 14)TRUE	0.843936298	0.05869183	0.000000e+00
I(RR.I == 0)TRUE	1.753026359	0.11883306	0.000000e+00
I(RR.I == 1)TRUE	0.909867448	0.18444952	8.199679e-07
I(RR.I == 2)TRUE	0.564555067	0.11074981	3.487220e-07
I(RR.I == 4)TRUE	0.027401674	0.06447239	6.708332e-01
I(RR.I == 5)TRUE	0.110883199	0.06213269	7.434569e-02
I(RR.I == 6)TRUE	0.313104741	0.06596504	2.090782e-06
I(RR.I == 7)TRUE	0.560365281	0.07503383	8.637535e-14
I(RR.I == 8)TRUE	0.656914833	0.09686310	1.235811e-11

```
I(RR.I == 9)TRUE          0.950571433 0.11458571 0.000000e+00
I(RR.I == 10)TRUE         1.018212485 0.13491449 4.751755e-14
I(RR.I == 11)TRUE         0.860472101 0.23311538 2.240955e-04
I(RR.I == 12)TRUE         1.343173157 0.23786732 1.668442e-08
I(RR.I == 13)TRUE         1.147765146 0.41706641 5.931278e-03
I(RR.I >= 14)TRUE         0.165684126 0.38987189 6.708649e-01
# Categories for decreased systolic blood pressure (0-49mmHg)
# was initially integrated into 1 category.
I(SBP.I %in% c(0:4))TRUE  2.895201311 0.09389737 0.000000e+00
I(SBP.I == 5)TRUE         1.845658318 0.17139869 0.000000e+00
I(SBP.I == 6)TRUE         1.569613042 0.10546774 0.000000e+00
I(SBP.I == 7)TRUE         1.427500533 0.09414590 0.000000e+00
I(SBP.I == 8)TRUE         1.093884740 0.08994293 0.000000e+00
I(SBP.I == 9)TRUE         0.736180043 0.08804793 0.000000e+00
I(SBP.I == 10)TRUE        0.529807054 0.08576413 6.701113e-10
I(SBP.I == 11)TRUE        0.304651035 0.08260371 2.268239e-04
I(SBP.I == 12)TRUE        0.212396872 0.07891211 7.120635e-03
I(SBP.I == 14)TRUE        0.088265754 0.07913580 2.647116e-01
I(SBP.I == 15)TRUE        0.168194168 0.08242884 4.132236e-02
I(SBP.I == 16)TRUE        0.258436968 0.08372471 2.027725e-03
I(SBP.I == 17)TRUE        0.271571532 0.09162897 3.043824e-03
I(SBP.I == 18)TRUE        0.364697223 0.09601877 1.464014e-04
I(SBP.I == 19)TRUE        0.552220201 0.10593870 1.889369e-07
I(SBP.I == 20)TRUE        0.620128808 0.11857982 1.724020e-07
I(SBP.I == 21)TRUE        0.821182907 0.13816442 2.858903e-09
I(SBP.I == 22)TRUE        0.656356280 0.17735753 2.158211e-04
I(SBP.I == 23)TRUE        1.123898100 0.21436786 1.605180e-07
I(SBP.I == 24)TRUE        1.195826833 0.27621443 1.506399e-05
I(SBP.I >= 25)TRUE        1.217612394 0.28119247 1.500727e-05
```

Step 3. Integration of adjunct categories of numeric variables

A logistic regression analysis was repeated after each modification on predictor partitioning. Adjunct partitions in a predictor was integrated if a category which was more distant from the reference (physiologically normal) category had lower regression coefficient in association with hospital mortality while it was theoretically greater than that nearer to the reference category. Logistic regression analyses were repeated until monotonic association of increase in regression coefficients and distant from the

reference category was achieved in all the predictor variables

R script and log for the step 3

```
# An R script for the step 3 was omitted.

# A R log for the step 3
# Categories for 3rd (16 to 19 year old) and 4th (20 to 24
# year old) age category was integrated into 1 category to
# maintain monotonic or U-shaped association explained in
# the step 3.
                             Estimate    std.error  p.value
(Intercept)                -5.471665391 0.11808381 0.000000e+00
I(Age.I %in% c(3:4))TRUE  0.005564763 0.10162149 9.563307e-01
I(Age.I == 6)TRUE          0.045223598 0.12253910 7.120930e-01
I(Age.I == 7)TRUE          0.025353696 0.11635110 8.275050e-01
I(Age.I == 8)TRUE          0.213005048 0.11825438 7.168679e-02
I(Age.I == 9)TRUE          0.354798316 0.11392457 1.847535e-03
I(Age.I == 10)TRUE         0.355516830 0.11250164 1.580634e-03
I(Age.I == 11)TRUE         0.483589002 0.10715107 6.441454e-06
I(Age.I == 12)TRUE         0.716452481 0.10263036 3.070877e-12
I(Age.I == 13)TRUE         0.889591529 0.09881058 0.000000e+00
I(Age.I == 14)TRUE         1.105178331 0.09840761 0.000000e+00
I(Age.I == 15)TRUE         1.252679455 0.09580954 0.000000e+00
I(Age.I == 16)TRUE         1.270204968 0.09741935 0.000000e+00
I(Age.I == 17)TRUE         1.310825913 0.10082805 0.000000e+00
I(Age.I >= 18)TRUE         1.307169517 0.10998038 0.000000e+00
I(GCS == 3)TRUE           4.177583101 0.06037804 0.000000e+00
[…]

# Age categories demonstrated U-shaped association with in-
# hospital mortality in reference to the 5th category (25 to 29
# year old).
                              estimate  std.error    p.value
(Intercept)                 -5.470360925 0.11799708 0.000000e+00
I(Age.I %in% c(3:4))TRUE    0.005051272 0.10170978 9.603913e-01
I(Age.I %in% c(6:7))TRUE    0.035266610 0.10406834 7.347063e-01
```

```
I(Age.I == 8)TRUE          0.211535518 0.11823931 7.363178e-02
I(Age.I %in% c(9:10))TRUE   0.354936405 0.09991705 3.832756e-04
I(Age.I == 11)TRUE         0.483705252 0.10708706 6.331827e-06
I(Age.I == 12)TRUE         0.713309910 0.10270765 3.971268e-12
I(Age.I == 13)TRUE         0.889032399 0.09896498 0.000000e+00
I(Age.I == 14)TRUE         1.102707372 0.09865013 0.000000e+00
I(Age.I == 15)TRUE         1.250898850 0.09597654 0.000000e+00
I(Age.I == 16)TRUE         1.267976535 0.09773844 0.000000e+00
I(Age.I >= 17)TRUE         1.303903751 0.09602657 0.000000e+00
I(GCS == 3)TRUE           4.176142669 0.06042779 0.000000e+00
[…]
```

Step 4. Integerize regression coefficient

Similar to step 3, adjunct partitions in a predictor was integrated to the nearest multiples of 0.67 (i.e. 0.67, 1.34, 2.01, ...). A 1-point increment in TRIAGES score is approximately equal to 0.67 increment in regression coefficient or odds ratio of 1.95 (TABLE2).

R script and log for the step 4

```
# An R script for the step 4 was omitted.

# A R log for the step 4
# The 3rd (16 to 19 year old) to 4th (20 to 24 year old) age
# category was similar regression coefficient around 0.00 and
# was integrated into 1 category (16 to 29 year old).
                          estimate    std.error  p.value
(Intercept)             -5.46688882 0.09713065 0.000000e+00
I(Age.I %in% c(6:7))TRUE   0.03183201 0.07852223 6.851990e-01
I(Age.I == 8)TRUE          0.20809696 0.09537542 2.913763e-02
I(Age.I %in% c(9:10))TRUE   0.35149568 0.07171387 9.637462e-07
I(Age.I == 11)TRUE         0.48026309 0.08257141 6.163098e-09
I(Age.I == 12)TRUE         0.70986509 0.07601195 0.000000e+00
I(Age.I == 13)TRUE         0.88558714 0.07126495 0.000000e+00
I(Age.I == 14)TRUE         1.09926013 0.07215084 0.000000e+00
```
