# Supplementary material for: Derivation and validation of an easy-to-compute trauma score that improves prognostication of mortality or the Trauma Rating Index in Age, Glasgow Coma Scale, Respiratory rate and Systolic blood pressure (TRIAGES) score
Source: Crit Care. 2019 Nov 21;23:365. doi: 10.1186/s13054-019-2636-x (PMC6868841; doi:10.1186/s13054-019-2636-x)
Supplement: Supplementary file 2 — Additional file 2: Table S1. Study variables included in outlier removal and multiple imputation. [file 13054_2019_2636_MOESM2_ESM.pdf]

Additional File 2: Table S1. Study variables included in outlier removal and multiple imputation

Variables	JTDB derivation cohort			JTDB validation cohort			CRASH-2 validation cohort		
	Naive	After outlier removal	After multiple imputation	Naive	After outlier removal	After multiple imputation	Naive	After outlier removal	After multiple imputation
Number of subjects	99867	99867	99867	110885	110885	110885	20197	20197	20197
Sex, N (%)									
Female	36684 (36.7)	36684 (36.7)	36690 (36.7)	42727 (38.5)	42727 (38.5)	42738 (38.5)	3269 (16.2)	3269 (16.2)	3269 (16.2)
Male	63167 (63.3)	63167 (63.3)	63177 (63.3)	68132 (61.4)	68132 (61.4)	68147 (61.5)	16927 (83.8)	16927 (83.8)	16928 (83.8)
Missing data	16 (0.0)	16 (0.0)	0 (0.0)	26 (0.0)	26 (0.0)	0 (0.0)	1 (0.0)	1 (0.0)	0 (0.0)
Age (year old)									
Median [IQR]	61 [39, 76]	61 [39, 76]	61 [39, 76]	63 [41, 78]	63 [41, 78]	63 [41, 78]	30 [24, 43]	30 [24, 43]	30 [24, 43]
Missing data	0 (0.0)	0 (0.0)	0 (0.0)	0 (0.0)	0 (0.0)	0 (0.0)	0 (0.0)	1 (0.0)	0 (0.0)
Year of injury onset, N (%)									
2000s (2004 to 2009)	23457 (23.5)	23457 (23.5)	23561 (23.6)	26656 (24.0)	26656 (24.0)	26816 (24.2)	20099 (99.5)	20099 (99.5)	20099 (99.5)
2010s (2010 to 2015)	75906 (76.0)	75906 (76.0)	76306 (76.4)	83581 (75.4)	83581 (75.4)	84069 (75.8)	98 (0.5)	98 (0.5)	98 (0.5)
Missing data	504 (0.5)	504 (0.5)	0 (0.0)	648 (0.6)	648 (0.6)	0 (0.0)	0 (0.0)	0 (0.0)	0 (0.0)
Cause of trauma, N (%)									
Accident	81831 (81.9)	81831 (81.9)	83368 (83.5)	94124 (84.9)	94124 (84.9)	96171 (86.7)			
Suicide	7538 (7.5)	7538 (7.5)	7919 (7.9)	5972 (5.4)	5972 (5.4)	6352 (5.7)			
Criminal	1701 (1.7)	1701 (1.7)	1792 (1.8)	1628 (1.5)	1628 (1.5)	1737 (1.6)			
Labour	6195 (6.2)	6195 (6.2)	6314 (6.3)	6003 (5.4)	6003 (5.4)	6132 (5.5)			
Other	446 (0.4)	446 (0.4)	474 (0.5)	468 (0.4)	468 (0.4)	492 (0.4)			
Missing data	2156 (2.2)	2156 (2.2)	0 (0.0)	2690 (2.4)	2690 (2.4)	0 (0.0)			
EtOH intoxicated, N (%)									
No	51173 (51.2)	51173 (51.2)	85189 (85.3)	60288 (54.4)	60288 (54.4)	95476 (86.1)			
Yes	9531 (9.5)	9531 (9.5)	14678 (14.7)	10282 (9.3)	10282 (9.3)	15409 (13.9)			
Missing data	39163 (39.2)	39163 (39.2)	0 (0.0)	40315 (36.4)	40315 (36.4)	0 (0.0)			
Situation of trauma									
Blunt injuries									
MVA, car passenger at driver seat, N (%)									
No	87593 (87.7)	87593 (87.7)	90000 (90.1)	97752 (88.2)	97752 (88.2)	100044 (90.2)			
Yes	8992 (9.0)	8992 (9.0)	9867 (9.9)	10174 (9.2)	10174 (9.2)	10841 (9.8)			
Missing data	3282 (3.3)	3282 (3.3)	0 (0.0)	2959 (2.7)	2959 (2.7)	0 (0.0)			
MVA, car passenger at navigator seat, N (%)									
No	94955 (95.1)	94955 (95.1)	98149 (98.3)	106094 (95.7)	106094 (95.7)	108964 (98.3)			
Yes	1630 (1.6)	1630 (1.6)	1718 (1.7)	1832 (1.7)	1832 (1.7)	1921 (1.7)			
Missing data	3282 (3.3)	3282 (3.3)	0 (0.0)	2959 (2.7)	2959 (2.7)	0 (0.0)			
MVA, car passenger at rear seat, N (%)									
No	95477 (95.6)	95477 (95.6)	98690 (98.8)	106725 (96.2)	106725 (96.2)	109583 (98.8)			
Yes	1108 (1.1)	1108 (1.1)	1177 (1.2)	1201 (1.1)	1201 (1.1)	1302 (1.2)			
Missing data	3282 (3.3)	3282 (3.3)	0 (0.0)	2959 (2.7)	2959 (2.7)	0 (0.0)			

Variables	JTDB derivation cohort			JTDB validation cohort			CRASH-2 validation cohort		
	Naive	After outlier removal	After multiple imputation	Naive	After outlier removal	After multiple imputation	Naive	After outlier removal	After multiple imputation
MVA, motorbike driver, N (%)									
No	84304 (84.4)	84304 (84.4)	87372 (87.5)	94448 (85.2)	94448 (85.2)	97175 (87.6)			
Yes	12281 (12.3)	12281 (12.3)	12495 (12.5)	13478 (12.2)	13478 (12.2)	13710 (12.4)			
Missing data	3282 (3.3)	3282 (3.3)	0 (0.0)	2959 (2.7)	2959 (2.7)	0 (0.0)			
MVA, bicycle passenger, N (%)									
No	89214 (89.3)	89214 (89.3)	92276 (92.4)	100746 (90.9)	100746 (90.9)	103531 (93.4)			
Yes	7371 (7.4)	7371 (7.4)	7591 (7.6)	7180 (6.5)	7180 (6.5)	7354 (6.6)			
Missing data	3282 (3.3)	3282 (3.3)	0 (0.0)	2959 (2.7)	2959 (2.7)	0 (0.0)			
MVA, pedestrian, N (%)									
No	89229 (89.3)	89229 (89.3)	92293 (92.4)	100296 (90.5)	100296 (90.5)	103085 (93.0)			
Yes	7356 (7.4)	7356 (7.4)	7574 (7.6)	7630 (6.9)	7630 (6.9)	7800 (7.0)			
Missing data	3282 (3.3)	3282 (3.3)	0 (0.0)	2959 (2.7)	2959 (2.7)	0 (0.0)			
MVA, other, N (%)									
No	95942 (96.1)	95942 (96.1)	99167 (99.3)	107523 (97.0)	107523 (97.0)	110451 (99.6)			
Yes	643 (0.6)	643 (0.6)	700 (0.7)	403 (0.4)	403 (0.4)	434 (0.4)			
Missing data	3282 (3.3)	3282 (3.3)	0 (0.0)	2959 (2.7)	2959 (2.7)	0 (0.0)			
Free fall, N (%)									
No	85005 (85.1)	85005 (85.1)	87963 (88.1)	98209 (88.6)	98209 (88.6)	100925 (91.0)			
Yes	11580 (11.6)	11580 (11.6)	11904 (11.9)	9717 (8.8)	9717 (8.8)	9960 (9.0)			
Missing data	3282 (3.3)	3282 (3.3)	0 (0.0)	2959 (2.7)	2959 (2.7)	0 (0.0)			
Fall on stairs, N (%)									
No	86053 (86.2)	86053 (86.2)	89068 (89.2)	94865 (85.6)	94865 (85.6)	97515 (87.9)			
Yes	10532 (10.5)	10532 (10.5)	10799 (10.8)	13061 (11.8)	13061 (11.8)	13370 (12.1)			
Missing data	3282 (3.3)	3282 (3.3)	0 (0.0)	2959 (2.7)	2959 (2.7)	0 (0.0)			
Fall, N (%)									
No	72786 (72.9)	72786 (72.9)	75622 (75.7)	76591 (69.1)	76591 (69.1)	79100 (71.3)			
Yes	23799 (23.8)	23799 (23.8)	24245 (24.3)	31335 (28.3)	31335 (28.3)	31785 (28.7)			
Missing data	3282 (3.3)	3282 (3.3)	0 (0.0)	2959 (2.7)	2959 (2.7)	0 (0.0)			
Machine related injury, N (%)									
No	95264 (95.4)	95264 (95.4)	98486 (98.6)	106571 (96.1)	106571 (96.1)	109455 (98.7)			
Yes	1321 (1.3)	1321 (1.3)	1381 (1.4)	1355 (1.2)	1355 (1.2)	1430 (1.3)			
Missing data	3282 (3.3)	3282 (3.3)	0 (0.0)	2959 (2.7)	2959 (2.7)	0 (0.0)			
Injury by object, N (%)									
No	95654 (95.8)	95654 (95.8)	98879 (99.0)	107011 (96.5)	107011 (96.5)	109915 (99.1)			
Yes	931 (0.9)	931 (0.9)	988 (1.0)	915 (0.8)	915 (0.8)	970 (0.9)			
Missing data	3282 (3.3)	3282 (3.3)	0 (0.0)	2959 (2.7)	2959 (2.7)	0 (0.0)			

Variables	JTDB derivation cohort			JTDB validation cohort			CRASH-2 validation cohort		
	Naive	After outlier removal	After multiple imputation	Naive	After outlier removal	After multiple imputation	Naive	After outlier removal	After multiple imputation
Crush injury, N (%)									
No	94923 (95.0)	94923 (95.0)	98133 (98.3)	106402 (96.0)	106402 (96.0)	109297 (98.6)			
Yes	1662 (1.7)	1662 (1.7)	1734 (1.7)	1524 (1.4)	1524 (1.4)	1588 (1.4)			
Missing data	3282 (3.3)	3282 (3.3)	0 (0.0)	2959 (2.7)	2959 (2.7)	0 (0.0)			
Train related injury, N (%)									
No	95989 (96.1)	95989 (96.1)	99208 (99.3)	107440 (96.9)	107440 (96.9)	110339 (99.5)			
Yes	596 (0.6)	596 (0.6)	659 (0.7)	486 (0.4)	486 (0.4)	546 (0.5)			
Missing data	3282 (3.3)	3282 (3.3)	0 (0.0)	2959 (2.7)	2959 (2.7)	0 (0.0)			
Sports injuries, N (%)									
No	95303 (95.4)	95303 (95.4)	98511 (98.6)	106235 (95.8)	106235 (95.8)	109112 (98.4)			
Yes	1282 (1.3)	1282 (1.3)	1356 (1.4)	1691 (1.5)	1691 (1.5)	1773 (1.6)			
Missing data	3282 (3.3)	3282 (3.3)	0 (0.0)	2959 (2.7)	2959 (2.7)	0 (0.0)			
Other injuries, N (%)									
No	93921 (94.0)	93921 (94.0)	97098 (97.2)	104611 (94.3)	104611 (94.3)	107450 (96.9)			
Yes	2664 (2.7)	2664 (2.7)	2769 (2.8)	3315 (3.0)	3315 (3.0)	3435 (3.1)			
Missing data	3282 (3.3)	3282 (3.3)	0 (0.0)	2959 (2.7)	2959 (2.7)	0 (0.0)			
Penetrating injuries									
Stab injuries, N (%)									
No	93277 (93.4)	93277 (93.4)	96420 (96.5)	104616 (94.3)	104616 (94.3)	107485 (96.9)			
Yes	3308 (3.3)	3308 (3.3)	3447 (3.5)	3310 (3.0)	3310 (3.0)	3400 (3.1)			
Missing data	3282 (3.3)	3282 (3.3)	0 (0.0)	2959 (2.7)	2959 (2.7)	0 (0.0)			
Gunshot, N (%)									
No	96547 (96.7)	96547 (96.7)	99821 (100.0)	107878 (97.3)	107878 (97.3)	110828 (99.9)			
Yes	38 (0.0)	38 (0.0)	46 (0.0)	48 (0.0)	48 (0.0)	57 (0.1)			
Missing data	3282 (3.3)	3282 (3.3)	0 (0.0)	2959 (2.7)	2959 (2.7)	0 (0.0)			
Impalement, N (%)									
No	96479 (96.6)	96479 (96.6)	99749 (99.9)	107818 (97.2)	107818 (97.2)	110764 (99.9)			
Yes	106 (0.1)	106 (0.1)	118 (0.1)	108 (0.1)	108 (0.1)	121 (0.1)			
Missing data	3282 (3.3)	3282 (3.3)	0 (0.0)	2959 (2.7)	2959 (2.7)	0 (0.0)			
Other, N (%)									
No	96479 (96.6)	96479 (96.6)	99749 (99.9)	107818 (97.2)	107818 (97.2)	110764 (99.9)			
Yes	106 (0.1)	106 (0.1)	118 (0.1)	108 (0.1)	108 (0.1)	121 (0.1)			
Missing data	3282 (3.3)	3282 (3.3)	0 (0.0)	2959 (2.7)	2959 (2.7)	0 (0.0)			
Type of trauma, N (%) *									
Blunt injury	92841 (93.0)	92841 (93.0)	95943 (96.1)	104110 (93.9)	104110 (93.9)	106932 (96.4)	11184 (55.4)	11184 (55.4)	11184 (55.4)
Penetrating injury	3600 (3.6)	3600 (3.6)	3745 (3.7)	3658 (3.3)	3658 (3.3)	3765 (3.4)	6549 (32.4)	6549 (32.4)	6549 (32.4)
Blunt and penetrating injury	144 (0.1)	144 (0.1)	178 (0.2)	158 (0.1)	158 (0.1)	188 (0.2)	2464 (12.2)	2464 (12.2)	2464 (12.2)
Missing data	3282 (3.3)	3282 (3.3)	1 (0.0)	2959 (2.7)	2959 (2.7)	1 (0.0)	0 (0.0)	0 (0.0)	0 (0.0)

Variables	JTDB derivation cohort			JTDB validation cohort			CRASH-2 validation cohort		
	Naive	After outlier removal	After multiple imputation	Naive	After outlier removal	After multiple imputation	Naive	After outlier removal	After multiple imputation
Vital signs at prehospital									
Systolic blood pressure (mmHg)									
Median [IQR]	134 [113, 157]	134 [113, 157]	132 [110, 155]	136 [116, 158]	136 [116, 158]	134 [112, 156]			
Missing data	30015 (30.1)	30016 (30.1)	0 (0.0)	28287 (25.5)	28288 (25.5)	0 (0.0)			
Heart rate (/minute)									
Median [IQR]	84 [72, 97]	84 [72, 97]	83 [71, 96]	84 [72, 96]	84 [72, 96]	83 [71, 96]			
Missing data	26396 (26.4)	26409 (26.4)	0 (0.0)	23532 (21.2)	23539 (21.2)	0 (0.0)			
Respiratory rate (/minute)									
Median [IQR]	20 [18, 24]	20 [18, 24]	20 [18, 24]	20 [18, 24]	20 [18, 24]	20 [18, 24]			
Missing data	33716 (33.8)	33811 (33.9)	0 (0.0)	31548 (28.5)	31574 (28.5)	0 (0.0)			
Japan Coma Scale, N (%)									
0	33397 (33.4)	33397 (33.4)	46770 (46.8)	43547 (39.3)	43547 (39.3)	57144 (51.5)			
1	12666 (12.7)	12666 (12.7)	16329 (16.4)	14260 (12.9)	14260 (12.9)	17693 (16.0)			
2	5589 (5.6)	5589 (5.6)	7084 (7.1)	6335 (5.7)	6335 (5.7)	7742 (7.0)			
3	4480 (4.5)	4480 (4.5)	5580 (5.6)	4601 (4.1)	4601 (4.1)	5576 (5.0)			
10	3837 (3.8)	3837 (3.8)	4769 (4.8)	4117 (3.7)	4117 (3.7)	4974 (4.5)			
20	1130 (1.1)	1130 (1.1)	1386 (1.4)	1120 (1.0)	1120 (1.0)	1342 (1.2)			
30	1342 (1.3)	1342 (1.3)	1640 (1.6)	1355 (1.2)	1355 (1.2)	1617 (1.5)			
100	2395 (2.4)	2395 (2.4)	2852 (2.9)	2336 (2.1)	2336 (2.1)	2731 (2.5)			
200	2552 (2.6)	2552 (2.6)	2969 (3.0)	2397 (2.2)	2397 (2.2)	2738 (2.5)			
300	9274 (9.3)	9274 (9.3)	10489 (10.5)	8363 (7.5)	8363 (7.5)	9326 (8.4)			
Missing data	23205 (23.2)	23205 (23.2)	0 (0.0)	22454 (20.2)	22454 (20.2)	0 (0.0)			
Vital signs on arrival at an emergency department									
Systolic blood pressure (mmHg)									
Median [IQR]	134 [112, 155]	134 [112, 155]	133 [112, 155]	135 [115, 156]	135 [115, 156]	135 [114, 156]	95 [80, 110]	95 [80, 110]	94 [80, 110]
Missing data	3633 (3.6)	3635 (3.6)	0 (0.0)	4014 (3.6)	4016 (3.6)	0 (0.0)	318 (1.6)	328 (1.6)	0 (0.0)
Heart rate (/minute)									
Median [IQR]	82 [70, 96]	82 [70, 96]	81 [70, 96]	82 [70, 95]	82 [70, 95]	82 [70, 95]	105 [90, 120]	105 [90, 120]	105 [90, 120]
Missing data	6574 (6.6)	6574 (6.6)	0 (0.0)	5594 (5.0)	5597 (5.0)	0 (0.0)	137 (0.7)	143 (0.7)	0 (0.0)
Respiratory rate (/minute)									
Median [IQR]	20 [16, 24]	20 [16, 24]	20 [16, 24]	20 [16, 24]	20 [16, 24]	20 [16, 24]	22 [20, 26]	22 [20, 26]	22 [20, 26]
Missing data	15555 (15.6)	15600 (15.6)	0 (0.0)	17539 (15.8)	17678 (15.9)	0 (0.0)	191 (0.9)	201 (1.0)	0 (0.0)
Temperature (Celcius)									
Median [IQR]	36.5 [35.9, 36.9]	36.5 [35.9, 36.9]	36.4 [35.9, 36.9]	36.5 [36.0, 36.9]	36.5 [36.0, 36.9]	36.5 [36.0, 36.9]			
Missing data	18829 (18.9)	19135 (19.2)	0 (0.0)	16094 (14.5)	16429 (14.8)	0 (0.0)			
Capillary refilling time (second)									
Median [IQR]							3 [2, 4]	3 [2, 4]	3 [2, 4]
Missing data							610 (3.0)	620 (3.1)	0 (0.0)

Variables	JTDB derivation cohort			JTDB validation cohort			CRASH-2 validation cohort		
	Naive	After outlier removal	After multiple imputation	Naive	After outlier removal	After multiple imputation	Naive	After outlier removal	After multiple imputation
Glasgow Coma Scale									
Eye, N (%)									
1	14271 (14.3)	14271 (14.3)	15777 (15.8)	12721 (11.5)	12721 (11.5)	14396 (13.0)	2652 (13.1)	2652 (13.1)	2914 (14.4)
2	1651 (1.7)	1651 (1.7)	2125 (2.1)	1584 (1.4)	1584 (1.4)	2060 (1.9)	1615 (8.0)	1615 (8.0)	1788 (8.9)
3	13872 (13.9)	13872 (13.9)	14925 (14.9)	13613 (12.3)	13613 (12.3)	14818 (13.4)	2740 (13.6)	2740 (13.6)	2986 (14.8)
4	60991 (61.1)	60991 (61.1)	67040 (67.1)	73346 (66.1)	73346 (66.1)	79611 (71.8)	10823 (53.6)	10823 (53.6)	12509 (61.9)
Missing data	9082 (9.1)	9082 (9.1)	0 (0.0)	9621 (8.7)	9621 (8.7)	0 (0.0)	2367 (11.7)	2367 (11.7)	0 (0.0)
Verbal, N (%)									
1	13809 (13.8)	13809 (13.8)	15652 (15.7)	12589 (11.4)	12589 (11.4)	14201 (12.8)	2567 (12.7)	2567 (12.7)	2853 (14.1)
2	3293 (3.3)	3293 (3.3)	3916 (3.9)	2966 (2.7)	2966 (2.7)	3501 (3.2)	1200 (5.9)	1200 (5.9)	1341 (6.6)
3	2410 (2.4)	2410 (2.4)	2705 (2.7)	2462 (2.2)	2462 (2.2)	2847 (2.6)	903 (4.5)	903 (4.5)	988 (4.9)
4	14246 (14.3)	14246 (14.3)	15382 (15.4)	15020 (13.5)	15020 (13.5)	16230 (14.6)	3160 (15.6)	3160 (15.6)	3360 (16.6)
5	56943 (57.0)	56943 (57.0)	62212 (62.3)	68157 (61.5)	68157 (61.5)	74105 (66.8)	9994 (49.5)	9994 (49.5)	11655 (57.7)
Missing data	9166 (9.2)	9166 (9.2)	0 (0.0)	9691 (8.7)	9691 (8.7)	0 (0.0)	2373 (11.7)	2373 (11.7)	0 (0.0)
Motor, N (%)									
1	9230 (9.2)	9230 (9.2)	10589 (10.6)	8059 (7.3)	8059 (7.3)	9144 (8.2)	788 (3.9)	788 (3.9)	892 (4.4)
2	825 (0.8)	825 (0.8)	1010 (1.0)	935 (0.8)	935 (0.8)	1231 (1.1)	607 (3.0)	607 (3.0)	679 (3.4)
3	572 (0.6)	572 (0.6)	709 (0.7)	625 (0.6)	625 (0.6)	825 (0.7)	658 (3.3)	658 (3.3)	723 (3.6)
4	3222 (3.2)	3222 (3.2)	3756 (3.8)	2966 (2.7)	2966 (2.7)	3620 (3.3)	1144 (5.7)	1144 (5.7)	1239 (6.1)
5	6135 (6.1)	6135 (6.1)	6900 (6.9)	6277 (5.7)	6277 (5.7)	7196 (6.5)	3009 (14.9)	3009 (14.9)	3255 (16.1)
6	70724 (70.8)	70724 (70.8)	76904 (77.0)	82332 (74.2)	82332 (74.2)	88868 (80.1)	11624 (57.6)	11624 (57.6)	13409 (66.4)
Missing data	9159 (9.2)	9159 (9.2)	0 (0.0)	9691 (8.7)	9691 (8.7)	0 (0.0)	2367 (11.7)	2367 (11.7)	0 (0.0)
Total									
Median [IQR]	15 [13, 15]	15 [13, 15]	15 [13, 15]	15 [14, 15]	15 [14, 15]	15 [13, 15]	15 [11, 15]	15 [11, 15]	15 [11, 15]
Missing data	9333 (9.3)	9333 (9.3)	0 (0.0)	9884 (8.9)	9884 (8.9)	0 (0.0)	23 (0.1)	23 (0.1)	0 (0.0)
Japan Coma Scale, N (%)									
0	38570 (38.6)	38570 (38.6)	49225 (49.3)	47427 (42.8)	47427 (42.8)	57637 (52.0)			
1	9901 (9.9)	9901 (9.9)	13531 (13.5)	12450 (11.2)	12450 (11.2)	15830 (14.3)			
2	4457 (4.5)	4457 (4.5)	6346 (6.4)	6118 (5.5)	6118 (5.5)	8027 (7.2)			
3	3395 (3.4)	3395 (3.4)	4892 (4.9)	4006 (3.6)	4006 (3.6)	5322 (4.8)			
10	5060 (5.1)	5060 (5.1)	7134 (7.1)	5199 (4.7)	5199 (4.7)	6925 (6.2)			
20	1107 (1.1)	1107 (1.1)	1528 (1.5)	1154 (1.0)	1154 (1.0)	1541 (1.4)			
30	1012 (1.0)	1012 (1.0)	1396 (1.4)	1060 (1.0)	1060 (1.0)	1434 (1.3)			
100	1969 (2.0)	1969 (2.0)	2589 (2.6)	1865 (1.7)	1865 (1.7)	2475 (2.2)			
200	2422 (2.4)	2422 (2.4)	3140 (3.1)	2256 (2.0)	2256 (2.0)	2947 (2.7)			
300	7988 (8.0)	7988 (8.0)	10085 (10.1)	6871 (6.2)	6871 (6.2)	8748 (7.9)			
Missing data	23986 (24.0)	23986 (24.0)	0 (0.0)	22479 (20.3)	22479 (20.3)	0 (0.0)			

Variables	JTDB derivation cohort			JTDB validation cohort			CRASH-2 validation cohort		
	Naive	After outlier removal	After multiple imputation	Naive	After outlier removal	After multiple imputation	Naive	After outlier removal	After multiple imputation
Abbreviated injury scale									
Region 1: Head, N (%)									
0	58180 (58.3)	58180 (58.3)	60246 (60.3)	62934 (56.8)	62934 (56.8)	65883 (59.4)			
1	4795 (4.8)	4795 (4.8)	4922 (4.9)	5190 (4.7)	5190 (4.7)	5425 (4.9)			
2	3956 (4.0)	3956 (4.0)	4032 (4.0)	4353 (3.9)	4353 (3.9)	4555 (4.1)			
3	11458 (11.5)	11458 (11.5)	11623 (11.6)	11837 (10.7)	11837 (10.7)	12600 (11.4)			
4	10643 (10.7)	10643 (10.7)	10935 (10.9)	11761 (10.6)	11761 (10.6)	13388 (12.1)			
5	7196 (7.2)	7196 (7.2)	7526 (7.5)	6982 (6.3)	6982 (6.3)	8366 (7.5)			
6	517 (0.5)	517 (0.5)	582 (0.6)	313 (0.3)	313 (0.3)	668 (0.6)			
Missing data	3122 (3.1)	3122 (3.1)	0 (0.0)	7515 (6.8)	7515 (6.8)	0 (0.0)			
Region 2: Face, N (%)									
0	80719 (80.8)	80719 (80.8)	83311 (83.4)	86446 (78.0)	86446 (78.0)	92618 (83.5)			
1	9164 (9.2)	9164 (9.2)	9436 (9.4)	9910 (8.9)	9910 (8.9)	10575 (9.5)			
2	6076 (6.1)	6076 (6.1)	6253 (6.3)	6398 (5.8)	6398 (5.8)	6797 (6.1)			
3	734 (0.7)	734 (0.7)	756 (0.8)	749 (0.7)	749 (0.7)	797 (0.7)			
4	107 (0.1)	107 (0.1)	111 (0.1)	93 (0.1)	93 (0.1)	99 (0.1)			
Missing data	3067 (3.1)	3067 (3.1)	0 (0.0)	7289 (6.6)	7289 (6.6)	0 (0.0)			
Region 3: Neck, N (%)									
0	95417 (95.5)	95417 (95.5)	98472 (98.6)	101990 (92.0)	101990 (92.0)	109277 (98.6)			
1	692 (0.7)	692 (0.7)	724 (0.7)	802 (0.7)	802 (0.7)	856 (0.8)			
2	219 (0.2)	219 (0.2)	229 (0.2)	277 (0.2)	277 (0.2)	298 (0.3)			
3	325 (0.3)	325 (0.3)	340 (0.3)	338 (0.3)	338 (0.3)	361 (0.3)			
4	66 (0.1)	66 (0.1)	70 (0.1)	53 (0.0)	53 (0.0)	57 (0.1)			
5	31 (0.0)	31 (0.0)	32 (0.0)	31 (0.0)	31 (0.0)	33 (0.0)			
6	0 (0.0)	0 (0.0)	0 (0.0)	2 (0.0)	2 (0.0)	3 (0.0)			
Missing data	3117 (3.1)	3117 (3.1)	0 (0.0)	7392 (6.7)	7392 (6.7)	0 (0.0)			
Region 4: Chest, N (%)									
0	67383 (67.5)	67383 (67.5)	69479 (69.6)	74978 (67.6)	74978 (67.6)	79180 (71.4)			
1	2625 (2.6)	2625 (2.6)	2705 (2.7)	2811 (2.5)	2811 (2.5)	3042 (2.7)			
2	2584 (2.6)	2584 (2.6)	2632 (2.6)	2487 (2.2)	2487 (2.2)	2715 (2.4)			
3	11493 (11.5)	11493 (11.5)	11770 (11.8)	11516 (10.4)	11516 (10.4)	12654 (11.4)			
4	9127 (9.1)	9127 (9.1)	9503 (9.5)	8345 (7.5)	8345 (7.5)	9312 (8.4)			
5	3056 (3.1)	3056 (3.1)	3248 (3.3)	2735 (2.5)	2735 (2.5)	3210 (2.9)			
6	454 (0.5)	454 (0.5)	530 (0.5)	397 (0.4)	397 (0.4)	772 (0.7)			
Missing data	3145 (3.1)	3145 (3.1)	0 (0.0)	7616 (6.9)	7616 (6.9)	0 (0.0)			

Variables	JTDB derivation cohort			JTDB validation cohort			CRASH-2 validation cohort		
	Naive	After outlier removal	After multiple imputation	Naive	After outlier removal	After multiple imputation	Naive	After outlier removal	After multiple imputation
Region 5: Abdomen, N (%)									
0	85480 (85.6)	85480 (85.6)	87926 (88.0)	91991 (83.0)	91991 (83.0)	97267 (87.7)			
1	1602 (1.6)	1602 (1.6)	1664 (1.7)	1766 (1.6)	1766 (1.6)	2065 (1.9)			
2	3972 (4.0)	3972 (4.0)	4173 (4.2)	3894 (3.5)	3894 (3.5)	4170 (3.8)			
3	3584 (3.6)	3584 (3.6)	3780 (3.8)	3842 (3.5)	3842 (3.5)	4092 (3.7)			
4	1684 (1.7)	1684 (1.7)	1841 (1.8)	1600 (1.4)	1600 (1.4)	1771 (1.6)			
5	400 (0.4)	400 (0.4)	461 (0.5)	392 (0.4)	392 (0.4)	524 (0.5)			
6	15 (0.0)	15 (0.0)	22 (0.0)	14 (0.0)	14 (0.0)	995 (0.9)			
Missing data	3130 (3.1)	3130 (3.1)	0 (0.0)	7386 (6.7)	7386 (6.7)	0 (0.0)			
Region 6: Spine, N (%)									
0	77552 (77.7)	77552 (77.7)	79004 (79.1)	84388 (76.1)	84388 (76.1)	90545 (81.7)			
1	1025 (1.0)	1025 (1.0)	1082 (1.1)	1612 (1.5)	1612 (1.5)	1717 (1.5)			
2	8173 (8.2)	8173 (8.2)	8335 (8.3)	8221 (7.4)	8221 (7.4)	8744 (7.9)			
3	5308 (5.3)	5308 (5.3)	5512 (5.5)	4829 (4.4)	4829 (4.4)	5160 (4.7)			
4	3216 (3.2)	3216 (3.2)	3356 (3.4)	3123 (2.8)	3123 (2.8)	3283 (3.0)			
5	1160 (1.2)	1160 (1.2)	1245 (1.2)	1081 (1.0)	1081 (1.0)	1129 (1.0)			
6	319 (0.3)	319 (0.3)	1332 (1.3)	282 (0.3)	282 (0.3)	306 (0.3)			
Missing data	3114 (3.1)	3114 (3.1)	0 (0.0)	7349 (6.6)	7349 (6.6)	0 (0.0)			
Region 7: Upper extremities, N (%)									
0	72244 (72.3)	72244 (72.3)	74675 (74.8)	79449 (71.6)	79449 (71.6)	85257 (76.9)			
1	6149 (6.2)	6149 (6.2)	6290 (6.3)	6564 (5.9)	6564 (5.9)	6953 (6.3)			
2	12799 (12.8)	12799 (12.8)	13112 (13.1)	12905 (11.6)	12905 (11.6)	13671 (12.3)			
3	5628 (5.6)	5628 (5.6)	5789 (5.8)	4709 (4.2)	4709 (4.2)	5004 (4.5)			
Missing data	3047 (3.1)	3047 (3.1)	0 (0.0)	7258 (6.5)	7258 (6.5)	0 (0.0)			
Region 8: Lower extremities, N (%)									
0	53053 (53.1)	53053 (53.1)	55223 (55.3)	57327 (51.7)	57327 (51.7)	61013 (55.0)			
1	5405 (5.4)	5405 (5.4)	5531 (5.5)	5847 (5.3)	5847 (5.3)	6315 (5.7)			
2	9729 (9.7)	9729 (9.7)	9920 (9.9)	9472 (8.5)	9472 (8.5)	10209 (9.2)			
3	24711 (24.7)	24711 (24.7)	25025 (25.1)	27562 (24.9)	27562 (24.9)	29212 (26.3)			
4	2136 (2.1)	2136 (2.1)	2255 (2.3)	1958 (1.8)	1958 (1.8)	2359 (2.1)			
5	1780 (1.8)	1780 (1.8)	1914 (1.9)	1445 (1.3)	1445 (1.3)	1777 (1.6)			
Missing data	3053 (3.1)	3053 (3.1)	0 (0.0)	7274 (6.6)	7274 (6.6)	0 (0.0)			

Variables	JTDB derivation cohort			JTDB validation cohort			CRASH-2 validation cohort		
	Naive	After outlier removal	After multiple imputation	Naive	After outlier removal	After multiple imputation	Naive	After outlier removal	After multiple imputation
Region 9: Surface, N (%)									
0	63013 (92.6)	63013 (92.6)	65005 (95.6)	69298 (89.5)	69298 (89.5)	73812 (95.4)			
1	2848 (4.2)	2848 (4.2)	2926 (4.3)	3314 (4.3)	3314 (4.3)	3504 (4.5)			
2	71 (0.1)	71 (0.1)	73 (0.1)	56 (0.1)	56 (0.1)	60 (0.1)			
3	8 (0.0)	8 (0.0)	8 (0.0)	8 (0.0)	8 (0.0)	9 (0.0)			
4	3 (0.0)	3 (0.0)	4 (0.0)	2 (0.0)	2 (0.0)	3 (0.0)			
5	4 (0.0)	4 (0.0)	4 (0.0)	9 (0.0)	9 (0.0)	10 (0.0)			
6	0 (0.0)	0 (0.0)	0 (0.0)	0 (0.0)	0 (0.0)	0 (0.0)			
Missing data	2074 (3.0)	2074 (3.0)	0 (0.0)	4711 (6.1)	4711 (6.1)	0 (0.0)			
Injury Severity Score									
Median [IQR]	13 [9, 21]	13 [9, 21]	13 [9, 22]	10 [9, 20]	10 [9, 20]	10 [9, 21]			
Missing data	3481 (3.5)	3481 (3.5)	0 (0.0)	8226 (7.4)	8226 (7.4)	0 (0.0)			
Outcome after emergency room, N (%)									
Died during emergency room	4610 (4.6)	4610 (4.6)	4723 (4.7)	3827 (3.5)	3827 (3.5)	4093 (3.7)			
Survived after emergency room	92132 (92.3)	92132 (92.3)	95144 (95.3)	97668 (88.1)	97668 (88.1)	106792 (96.3)			
Missing data	3125 (3.1)	3125 (3.1)	0 (0.0)	9390 (8.5)	9390 (8.5)	0 (0.0)			
Outcome after hospitalization, N (%)									
Died during hospitalization	11622 (11.6)	11622 (11.6)	12473 (12.5)	9877 (8.9)	9877 (8.9)	11651 (10.5)	3085 (15.3)	3085 (15.3)	3085 (15.3)
Survived after hospitalization	81984 (82.1)	81984 (82.1)	87394 (87.5)	87371 (78.8)	87371 (78.8)	99234 (89.5)	17112 (84.7)	17112 (84.7)	17112 (84.7)
Missing data	6261 (6.3)	6261 (6.3)	0 (0.0)	13637 (12.3)	13637 (12.3)	0 (0.0)	0 (0.0)	0 (0.0)	0 (0.0)

Descriptive statistics of all the studied variables, which were included in multiple imputation after outlier removal, were summarized as those before multiple imputation (naive), those after outlier removal by robust regression and those after multiple imputation by chained equation, across the 3 datasets included in this study. Continuous variables were displayed as median with interquartile range (25th - 75th percentile). Categorical and ordered variables were displayed as count with percentage.; *, Type of trauma was included in multiple imputation and reassessed from situation of trauma after multiple imputation.; JTDB, the Japan Trauma Databank; CRASH-2, Clinical Randomisation of Antifibrinolytics in Significant Hemorrhage - 2; IQR, interquartile range; EtOH, ethanol; MVA, motor vehicle accident
