# Supplementary material for: Derivation and validation of an easy-to-compute trauma score that improves prognostication of mortality or the Trauma Rating Index in Age, Glasgow Coma Scale, Respiratory rate and Systolic blood pressure (TRIAGES) score
Source: Crit Care. 2019 Nov 21;23:365. doi: 10.1186/s13054-019-2636-x (PMC6868841; doi:10.1186/s13054-019-2636-x)
Supplement: Supplementary file 3 — Additional file 3: Table S2. Partitioning with assigned score values of physiological parameters included in the tested trauma scores. [file 13054_2019_2636_MOESM3_ESM.pdf]

Additional File 3: Table S2. Partitioning with assigned score values of physiological parameters included in the tested trauma scores.

	TRIAGES score		RTS		MGAP score	
Variables	Range	assigned score	Range	assigned score	Range	assigned score
Glasgow coma scale						
	3	6	3	0.0000	3	3
	4	5	4–5	0.9368	4	4
					5	5
	5–7	4			6	6
			6–8	1.8736	7	7
					8	8
					9	9
	8–11	3			10	10
			9–12	2.8104	11	11
	12–13	2			12	12
					13	13
	14	1	13–15	3.7472	14	14
	15	0			15	15
Systolic blood pressure, mmHg						
	0–49	4	0	0.0000	0–59	0
			1–49	0.7326		
	50–79	2	50–75	1.4652		
			76–89	2.1978	60–120	3
	80–99	1				
	100–199	0	90+	2.9304	121+	5
	200+	1				
Respiratory rate, /minute						
	0–3	2	0	0.0000	Not used	
			1–5	0.2908		
	4–11	1	6–9	0.5816		
	12–27	0	10–29	1.1632		
	28+	1	30+	0.8724		
Age, year old						
	16–54	0	Not used		0–59	5
	55–74	1				
	75+	2			60+	0
Mechanism of Injury						
	Not used		Not used		Blunt	4
					Penetrating	0

TRIAGES score, the Trauma Rating Index in Age, the Glasgow coma scale, Respiratory rate, and Systolic blood pressure score; RTS, the Revised Trauma Score; MGAP score, Mechanism, the Glasgow coma scale, Age, and arterial Pressure score
