# Supplementary material for: Derivation and validation of an easy-to-compute trauma score that improves prognostication of mortality or the Trauma Rating Index in Age, Glasgow Coma Scale, Respiratory rate and Systolic blood pressure (TRIAGES) score
Source: Crit Care. 2019 Nov 21;23:365. doi: 10.1186/s13054-019-2636-x (PMC6868841; doi:10.1186/s13054-019-2636-x)
Supplement: Supplementary file 4 — Additional file 4: Figure S1. Comparison of prognostic accuracies for in-hospital mortality in the studied trauma scores. [file 13054_2019_2636_MOESM4_ESM.pdf]

Additional File 4: Figure S1.

Comparison of prognostic accuracies for in-hospital mortality in the studied trauma scores

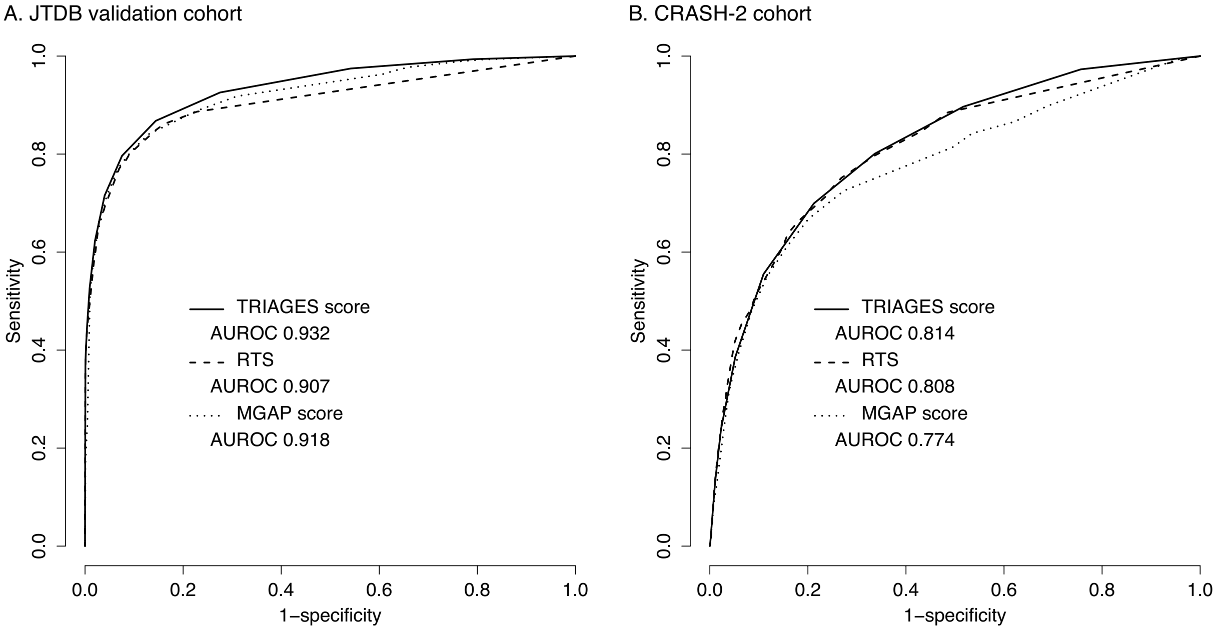

Prognostic accuracy for in-hospital mortality with the studied trauma scores are displayed for the JTDB validation cohort (A) and CRASH-2 cohort (B). JTDB, the Japan Trauma Databank; CRASH-2 Clinical Randomisation of Antifibrinolytics in Significant Hemorrhage-2; TRIAGES score, trauma rating index in age, Glasgow Coma Scale, respiratory rate, and systolic blood pressure score; RTS, the revised trauma score; MGAP score, mechanism, Glasgow Coma Scale, age and arterial pressure score; AUROC, area under curve.
