# Supplementary material for: Derivation and validation of an easy-to-compute trauma score that improves prognostication of mortality or the Trauma Rating Index in Age, Glasgow Coma Scale, Respiratory rate and Systolic blood pressure (TRIAGES) score
Source: Crit Care. 2019 Nov 21;23:365. doi: 10.1186/s13054-019-2636-x (PMC6868841; doi:10.1186/s13054-019-2636-x)
Supplement: Supplementary file 5 — Additional file 5: Figure S2. Calibration plots of the studied trauma scores. [file 13054_2019_2636_MOESM5_ESM.pdf]

Additional File 5: Figure S2. Calibration plots of the studied trauma scores

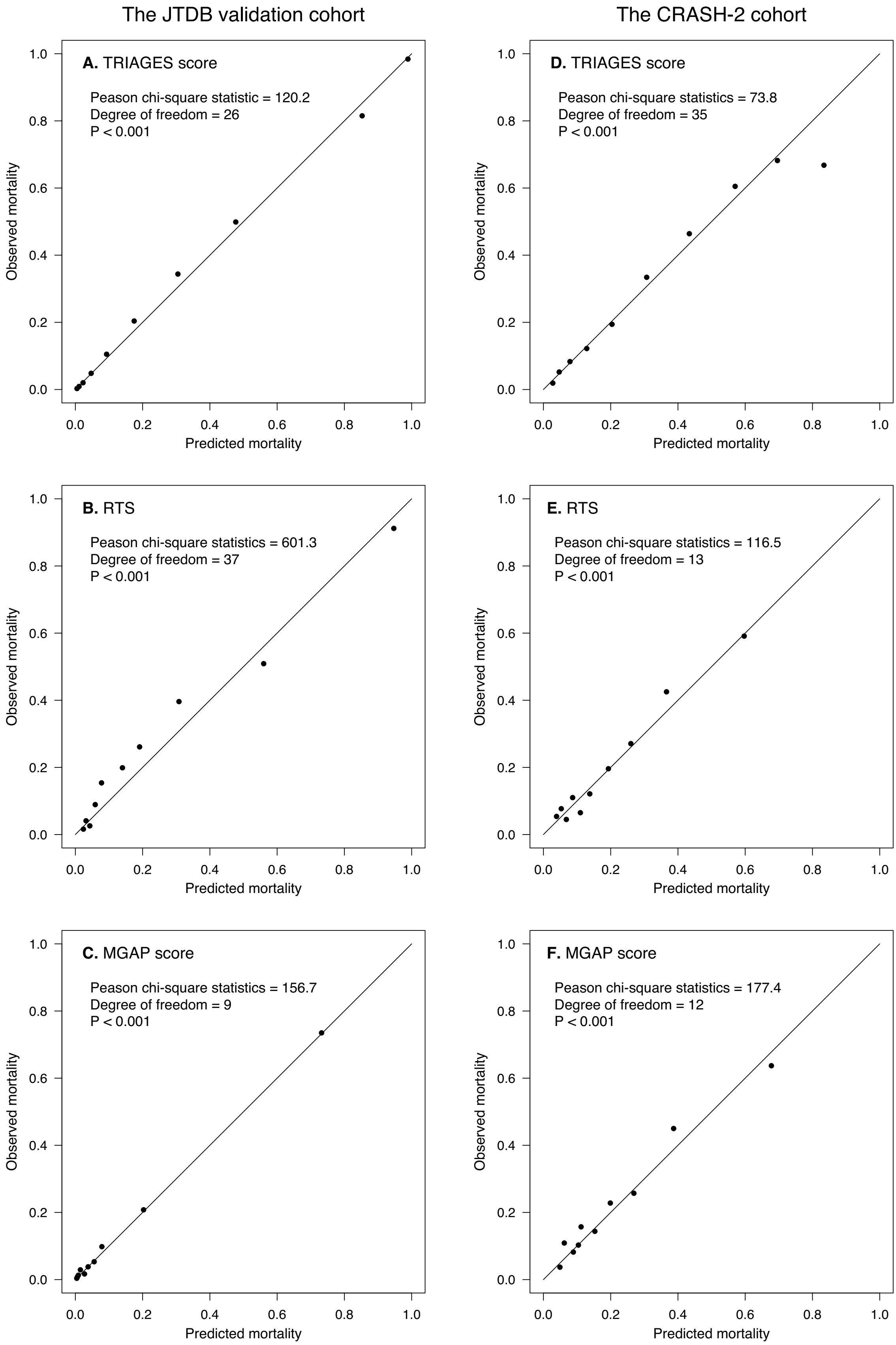

Calibration plot between predicted mortality and observed mortality of the tested scores was estimated on the JTDB validation (A-C) and CRASH-2 cohort (D-F). Smallest degree of freedom was used in each the analysis in order to do Hosmer-Lemeshow goodness of fit test with 10 groups. JTDB, the Japan Trauma Databank; CRASH-2 Clinical Randomisation of Antifibrinolytics in Significant Hemorrhage-2; TRIAGES score, trauma rating index in age, Glasgow Coma Scale, respiratory rate, and systolic blood pressure score; RTS, the revised trauma score; MGAP, mechanism, Glasgow Coma Scale, age and arterial pressure score
