# Supplementary material for: Derivation and validation of an easy-to-compute trauma score that improves prognostication of mortality or the Trauma Rating Index in Age, Glasgow Coma Scale, Respiratory rate and Systolic blood pressure (TRIAGES) score
Source: Crit Care. 2019 Nov 21;23:365. doi: 10.1186/s13054-019-2636-x (PMC6868841; doi:10.1186/s13054-019-2636-x)
Supplement: Supplementary file 6 — Additional file 6: Table S3. Comparisons of diagnostic indices across the validation cohorts. [file 13054_2019_2636_MOESM6_ESM.pdf]

Additional File 6: Table S3. Comparisons of diagnostic indices across the validation cohorts

Scores with threshold for the test positive	Sensitivity	Specificity	Positive predictive value	Negative predictive value
The JTDB validation cohort				
The best threshold				
TRIAGES score ≥5	0.796	0.925	0.544	0.975
RTS ≤6.8174	0.803	0.907	0.494	0.976
MGAP score ≤20	0.806	0.906	0.482	0.977
Threshold at sensitivity >0.9				
TRIAGES score ≥3	0.926	0.725	0.276	0.989
RTS ≤7.5500	0.887	0.772	0.306	0.984
MGAP score ≤23	0.919	0.690	0.244	0.987
CRASH-2 cohort				
The best threshold				
TRIAGES score ≥5	0.555	0.890	0.464	0.921
RTS ≤6.8174	0.751	0.731	0.324	0.945
MGAP score ≤20	0.611	0.842	0.404	0.926
Threshold at sensitivity >0.9				
TRIAGES score ≥3	0.801	0.663	0.289	0.951
RTS ≤7.5500	0.885	0.514	0.237	0.963
MGAP score ≤23	0.725	0.726	0.316	0.938

Diagnostic indices including sensitivity, specificity and positive and negative predictive values were estimated using the best threshold by Youden index or threshold at sensitivity >0.9 on the JTDB derivation cohort. JTDB, the Japan Trauma Databank; CRASH-2 Clinical Randomisation of Antifibrinolytics in Significant Hemorrhage-2; TRIAGES score, trauma rating index in age, Glasgow Coma Scale, respiratory rate, and systolic blood pressure score; RTS, the revised trauma score; MGAP, mechanism, Glasgow Coma Scale, age and arterial pressure score
