# Supplementary material for: Derivation and validation of an easy-to-compute trauma score that improves prognostication of mortality or the Trauma Rating Index in Age, Glasgow Coma Scale, Respiratory rate and Systolic blood pressure (TRIAGES) score
Source: Crit Care. 2019 Nov 21;23:365. doi: 10.1186/s13054-019-2636-x (PMC6868841; doi:10.1186/s13054-019-2636-x)
Supplement: Supplementary file 7 — Additional file 7: Table S4. Comparison of diagnostic accuracy of the tested scores stratified by age. [file 13054_2019_2636_MOESM7_ESM.pdf]

**Additional File 7: Table S4. Comparison of diagnostic accuracy of the tested scores stratified by age.**

| Subgroups | AUROC | | | Bootstrap comparisons | | | |
|---|---|---|---|---|---|---|---|
| | TRIAGES score | RTS | MGAP score | TRIAGES score versus RTS | | TRIAGES score versus MGAP score | |
| | | | | Difference [95%CI] | *P* | Difference [95%CI] | *P* |
| 16-39 year old | | | | | | | |
| JTDB validation cohort (N=18,914) | 0.965 | 0.963 | 0.957 | 0.002 [-0.001, 0.004] | 0.212 | 0.008 [0.005, 0.011] | <0.001 |
| CRASH-2 cohort (N=13,279) | 0.821 | 0.823 | 0.778 | -0.001 [-0.006, 0.004] | 0.606 | 0.043 [0.034, 0.052] | <0.001 |
| 40-59 year old | | | | | | | |
| JTDB validation cohort (N=16,886) | 0.949 | 0.945 | 0.940 | 0.004 [0.001, 0.008] | 0.024 | 0.010 [0.006, 0.013] | <0.001 |
| CRASH-2 cohort (N=4,781) | 0.785 | 0.786 | 0.747 | -0.001 [-0.011, 0.008] | 0.785 | 0.038 [0.025, 0.051] | <0.001 |
| 60-79 year old | | | | | | | |
| JTDB validation cohort (N=25,665) | 0.919 | 0.903 | 0.913 | 0.016 [0.012, 0.020] | <0.001 | 0.006 [0.003, 0.009] | <0.001 |
| CRASH-2 cohort (N=1,293) | 0.776 | 0.780 | 0.758 | -0.003 [-0.019, 0.013] | 0.688 | 0.019 [0.000, 0.038] | 0.052 |
| 80+ year old | | | | | | | |
| JTDB validation cohort (N=15,297) | 0.866 | 0.841 | 0.863 | 0.025 [0.018, 0.032] | <0.001 | 0.003 [-0.002, 0.008] | 0.211 |
| CRASH-2 cohort (N=130) | 0.813 | 0.797 | 0.780 | 0.014 [-0.032, 0.067] | 0.573 | 0.032 [-0.028, 0.093] | 0.295 |

Point estimation with 95%Cis and P-values of the metrics of score performance were computed by bootstrap estimation repeated for 20,000 times (800 times per each the dataset if bootstrapping was performed on multiply imputed datasets). AUROC, area under the receiver operating characteristics curve; TRIAGES score, trauma rating index in age, Glasgow Coma Scale, respiratory rate, and systolic blood pressure score; RTS, the revised trauma score; MGAP, mechanism, Glasgow Coma Scale, age and arterial pressure score; 95% CI, 95% confidence interval; JTDB, the Japan Trauma Databank; CRASH-2, Clinical Randomization of Antifibrinolytics in Significant Hemorrhage-2
